# Potassium, not lepidimoide, is the principal ‘allelochemical’ of cress-seed exudate that promotes amaranth hypocotyl elongation

**DOI:** 10.1093/aob/mcx081

**Published:** 2017-07-11

**Authors:** Stephen C Fry

**Affiliations:** 1 The Edinburgh Cell Wall Group, Institute of Molecular Plant Sciences, The University of Edinburgh, Daniel Rutherford Building, The King’s Buildings, Max Born Crescent, Edinburgh EH9 3BF, UK

**Keywords:** Allelopathy, amaranth (*Amaranthus caudatus*), cress (*Lepidium sativum*), hypocotyl elongation, root growth, lepidimoic acid, lepidimoide, potassium, rhamnogalacturonan-I

## Abstract

**Background and Aims** Imbibed cress (*Lepidium sativum* L.) seeds exude ‘allelochemicals’ that promote excessive hypocotyl elongation and inhibit root growth in neighbouring competitors, e.g. amaranth (*Amaranthus caudatus* L.) seedlings. The major hypocotyl promoter has recently been shown not to be the previously suggested acidic disaccharide, lepidimoic acid (LMA), a fragment of the pectic polysaccharide domain rhamnogalacturonan-I. The nature of the hypocotyl promoter has now been re-assessed.

**Methods** Low-molecular weight cress-seed exudate (LCSE) was fractionated by high-voltage electrophoresis, and components with different charge:mass ratios were tested for effects on dark-grown amaranth seedlings. Further samples of LCSE were size-fractionated by gel permeation chromatography, and active fractions were analysed electrophoretically.

**Key Results** The LCSE strongly promoted amaranth hypocotyl elongation. The active principle was hydrophilic and, unlike LMA, stable to hot acid. After electrophoresis at pH 6·5, the only fractions that strongly promoted hypocotyl elongation were those with a very high positive charge:mass ratio, migrating towards the cathode 3–4 times faster than glucosamine. Among numerous naturally occurring cations tested, the only one with such a high mobility was potassium. K^+^ was present in LCSE at approx. 4 mm, and pure KCl (1–10 mm) strongly promoted amaranth hypocotyl elongation. No other cation tested (including Na^+^, spermidine and putrescine) had this effect. The peak of bioactivity from a gel permeation chromatography column exactly coincided with the peak of K^+^.

**Conclusions** The major ‘allelopathic’ substance present in cress-seed exudate that stimulates hypocotyl elongation in neighbouring seedlings is the inorganic cation, K^+^, not the oligosaccharin LMA.

## INTRODUCTION

In model experiments, cress (*Lepidium sativum* L.) seeds adversely affect the growth of neighbouring, potentially competing, ‘receiver seedlings’ such as amaranth (*Amaranthus caudatus* L.) – an effect that has been described as allelopathic (Hasegawa *et al.*, 1992; Yamada *et al.*, 2007; Iqbal and Fry, 2012). Substances exuded by the cress seeds inhibit root growth and unduly increase the length:girth ratio of the hypocotyl in amaranth, leading to weakened receiver seedlings. Iqbal and Fry (2012) showed that the cress-seed exudate primarily targets cell expansion in the amaranth hypocotyl in a manner superficially resembling that of a gibberellin. Any effect on cell division was too small, or in the wrong direction, to account for the growth response. The factor is already present in dry cress seeds, and is progressively released during imbibition, even from heat-killed seeds (Iqbal and Fry, 2012).

It is possible that such biological effects raise the chances of cress seedling establishment by weakening potentially competing neighbours. Whether or not this interpretation is correct, it is of interest to characterize further the nature and production of growth-regulating active principle(s) exuded by seeds.

The effects of cress seed(ling)s on neighbouring receiver seedlings were first reported by Hasegawa and colleagues, and attributed by them to an allelochemical, namely lepidimoic acid (LMA; here taken to include its sodium salt, originally named ‘lepidimoide’), which is released by roots and by imbibed seeds of cress (Hasegawa *et al.*, 1992) and other plant species (Yamada *et al.*, 1995). LMA [4-deoxy-β-l-*threo*-hex-4-enopyranuronosyl-(1→2)-l-rhamnose] is an unsaturated acidic disaccharide, probably formed *in vivo* by the action of a lyase on the pectic polysaccharide domain, rhamnogalacturonan-I. The acid and its sodium salt, which are undoubtedly interconvertible *in vivo*, were reported to exert comparable biological effects (Yamada *et al.*, 1996). LMA thus appeared to be an interesting example of an oligosaccharin (biologically active oligosaccharide: Darvill *et al.*, 1992; Fry *et al.*, 1993; Kollárová *et al.*, 2005; Field, 2009; Cabrera *et al.*, 2013) that is chemically related to rhamnogalacturonan-I (Fry *et al.*, 1993) and also functions as an allelochemical. Hasegawa *et al.* (1992) first reported that 1 mm LMA promotes amaranth hypocotyl elongation 5-fold, and that even at 3 μm it has a 1·5-fold effect. They also reported that LMA >100 μm inhibits amaranth root growth. In later work, e.g. Yamada *et al.* (1996), promotion of amaranth hypocotyl elongation was reported to require an LMA concentration of at least 300 μm, which caused only an approx. 1·2-fold promotion; and root inhibition was not discussed.

Iqbal *et al.* (2016) recently confirmed that the active principle from cress seeds is of low molecular weight and that LMA is indeed present in cress-seed exudate. However, pure LMA at 360 μm evoked only a slight (1·15-fold) promotion of amaranth hypocotyl elongation; it also caused a 1·1-fold promotion of root growth (Iqbal *et al.*, 2016), contradicting the proposal (Hasegawa *et al.*, 1992) that LMA is a root growth inhibitor and serves as the major allelochemical of cress-seed exudate. A second acidic disaccharide, β-d-xylopyranosyl-(1→3)-d-galacturonic acid, likely to be a hydrolysis product of another pectic domain, xylogalacturonan, was also discovered in cress-seed exudate and found to exert, at 740 μm, minor biological effects similar to those of LMA (Iqbal *et al.*, 2016). Thus, the slight ‘allelochemical’ effects of LMA are not tightly dependent on its chemical structure. Furthermore, it was found that the major hypocotyl-stimulating factor present in cress-seed exudate failed to co-migrate with authentic LMA on high-voltage paper electrophoresis (HVPE), and failed to co-elute exactly with it during gel permeation chromatography (Iqbal *et al.*, 2016). It was concluded that the major active principle of cress-seed exudate remained unidentified. Perhaps the most puzzling observation was that the great majority of the biological activity was lost during HVPE, a highly effective method for purifying LMA. In the present work, the cress-seed ‘allelochemical’ has been re-investigated and found to be a cation with a very high charge:mass ratio.

## MATERIALS AND METHODS

### Materials

Cress seeds (*Lepidium sativum*) were from Sutton Seeds, Paignton, UK. Sterile 5-cm plastic Petri dishes were from Sterilin Ltd, Caerphilly, UK. Volatile electrophoresis buffers were from Fisher Scientific, Loughborough, UK. Filter paper discs (47 mm; Whatman No. 1), chromatography paper (Whatman No. 1 or 3) and general laboratory chemicals were from Sigma-Aldrich, Poole, UK.

### Preparation of low-molecular weight cress-seed exudate (LCSE)

Low-molecular weight cress-seed exudate was prepared as described by Iqbal *et al.* (2016). In brief, imbibed but ungerminated cress seeds (5 g d. wt) were placed in a dialysis sac with a total of 100 mL of water (about 50 mL inside the sac and 50 mL outside) for 48 h at 4 °C. The external solution (LCSE; approx. 50 mL; total dissolved solids approx. 1·6 mg mL^–1^) was filtered through filter paper and stored frozen.

### Properties of LCSE

In a study of the physical properties of the active principle(s) in LCSE, 13 independent preparations of LCSE were isolated. Identical 1-mL portions of each preparation were taken. One was simply frozen (and thawed when all the other samples were ready). Five other portions, (*a*)–(*e*), of each of the 13 preparations were treated respectively as follows. (*a*) Drying: the sample was dried *in vacuo* in a SpeedVac. (*b*) Cold acid: trifluoroacetic acid (TFA) was added to 1·2 m and incubated at room temperature for 30 min, then dried *in vacuo*. (*c*) Hot acid: as (*b*) but incubated at 120 °C for 30 min. (*d*) Solvent partitioning: TFA was added to 0·13 m, then the acidified aqueous solution was shaken with an equal volume of ethyl acetate, the two phases (ethyl acetate and H_2_O) were separated and each phase was dried *in vacuo*; a ‘solvents-only’ control [i.e. without LCSE but otherwise the same as (*d*)] was also prepared as a check that the TFA and ethyl acetate had been successfully removed by the drying step. (*e*) Ashing: further 1-mL aliquots were dried in Pyrex tubes which were then heated for 10 min at approx. 700 °C (in the hottest part of a Bunsen burner flame) or for 3 min at approx. 400 °C in a milder flame.

Each portion was then re-dissolved in 1 mL of water, and the solutions were applied to amaranth seeds as described below.

### Allelochemical bioassay

The solution (1 mL) to be tested for allelopathic activity was pipetted onto two 4·7-cm discs of Whatman No. 1 filter paper in a 5-cm plastic Petri dish, then ten amaranth seeds were placed (well spaced) on the paper. The lids were sealed with Parafilm and the dishes incubated in the dark at 25 °C for 4·5 d. The seedlings were then submerged for 10 min in 5 mL of a staining solution [0·01 % (w/v) aniline blue in 5 % (v/v) acetic acid (Long *et al.*, 2008)], rinsed in water, arranged on an acetate overhead-projector sheet on a background of graph paper, and scanned. The roots stain blue but the cuticularized hypocotyls remain white, facilitating the demarcation between the two organs. The seedlings were straightened by pulling for a short distance along the wet sheet of acetate. Hypocotyls were then measured from the hook to the junction with the root; the whole tap roots (there were no laterals) were also measured.

### High-voltage paper electrophoresis (HVPE)

High-voltage paper electrophoresis was conducted on 57 cm long sheets of Whatman No. 1 or No. 3 paper in volatile buffers [pyridine/acetic acid/H_2_O (33:1:300 v/v/v, pH 6·5) and formic acid/acetic acid/H_2_O (1:4:45, v/v/v, pH 2·0); voltages and times as specified in individual experiments], then dried to remove the buffers (Fry, 2011). When the separated zones were to be bioassayed, the paper was dried, dipped through acetone/methanol (2:1) and re-dried, and this cycle was repeated several times; strips of the paper were then eluted with water, and the eluate was dried *in vacuo* and re-dissolved in water.

Compounds on paper electrophoretograms were stained with aniline hydrogen phthalate (for reducing sugars), AgNO_3_ (for total sugars) and ninhydrin (for amino acids); the methods are summarized by Fry (2000). For detection of inorganic cations and anions, the paper was dipped through acetone/methanol as above, then quickly dipped through an indicator solution (ethanol containing 0·4 g L^–1^ bromophenol blue and 0·4 mL L^–1^ collidine) and hung to dry for about 15 min.

## RESULTS

### Cress seeds affect the growth of neighbouring amaranth seedlings

**Fig. 1.**
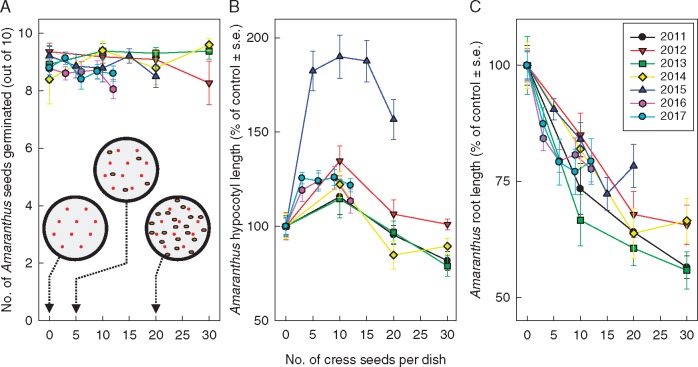
Cress seed(ling)s promote hypocotyl elongation and inhibit root growth in neighbouring amaranth seedlings. In each 5-cm Petri dish, ten (dry) amaranth seeds were sown, together with 0–30 (dry) cress seeds, as shown schematically in (A). Seeds of the two species were mingled, but no two seeds touched each other. After 4 d incubation, the number of amaranth seeds germinated was recorded (A), and organs of the amaranth seedlings were measured (B, hypocotyls; C, roots). The experiment was conducted in seven consecutive years, each time with fresh batches of seeds. Error bars show the inter-plate s.e. for each year; the number of Petri dishes (*n*) was 15, 11, 13, 10, 14, 18 and 16 for the years 2011–2017, respectively. In the cress-free controls, the mean amaranth hypocotyl lengths in the 7 years were, respectively, 16·5, 15·7, 10·4, 18·8, 12·1, 15·3 and 15·0 mm, and the mean root lengths were 29·4, 26·9, 24·7, 29·6, 31·8, 35·5 and 28·8 mm.

In view of the negligible effect of purified LMA on amaranth seedling growth (Iqbal *et al.*, 2016), the reported allelochemical effect of cress seeds, as potential allelochemical donors, on amaranth (allelochemical receiver) was re-tested. On replicate Petri dishes, ten amaranth seeds were sown along with various numbers (0–30) of cress seeds. The presence of cress had no effect on amaranth germination (mean 8·93 ± 0·09 germinated out of ten; Fig. 1A) but consistently inhibited root elongation (Fig. 1C). In contrast, a moderate density of cress seeds increased the elongation of amaranth hypocotyls (Fig. 1B). These effects were consistently observed in repeat experiments, conducted over 7 years, each time with a new batch of seeds, supporting earlier observations (Hasegawa *et al.*, 1992; Yamada *et al.*, 2007; Iqbal and Fry, 2012).

**Fig. 2.**
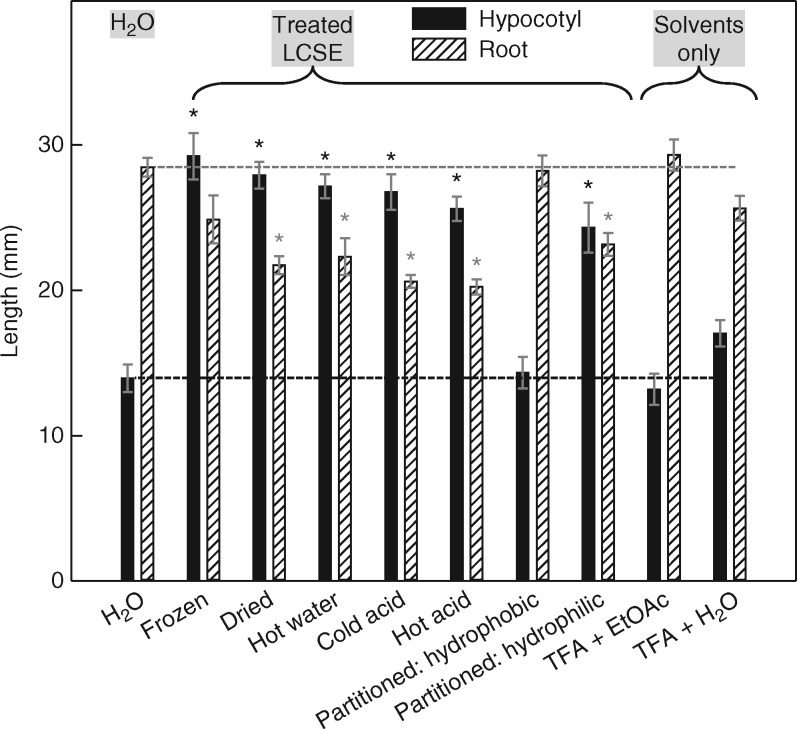
Imbibing cress seeds exude low-molecular-weight, heat-stable, acid-stable, hydrophilic substance(s) that promote hypocotyl elongation and inhibit root growth in neighbouring amaranth seedlings. Thirteen independent preparations of LCSE (substances exuded by imbibed cress seeds, and small enough to pass through a dialysis membrane) were isolated. Six identical portions of each preparation were treated as shown on the *x*-axis, then (except in the ‘frozen’ portion) dried and re-dissolved in the original volume of water. ‘Acid’ treatments were with 1·2 m trifluoroacetic acid (TFA) at 20 or 120 °C for 30 min. Solvent partitioning was between ethyl acetate (EtOAc; hydrophobic) and slightly acidified water (hydrophilic). ‘Solvent only’ samples (i.e. without LCSE) were also dried, and any residue was then re-dissolved in water as a check that the TFA and/or EtOAc had been successfully removed. The variously treated aliquots of LCSE were then used as media for the amaranth seed bioassay. There was a negligible effect on germination (mean 88–98 % germination in the ten treatments, each treatment replicated in 13 Petri dishes). After 4 d, the amaranth seedlings were measured (solid bars, hypocotyl; hatched bars, root; error bars show the inter-dish s.e.; *n* = 11–13]; *significantly different (*P* < 0·001) from the H_2_O-only control. Dashed lines: hypocotyl and root lengths obtained with pure water as the medium.

The seedlings were adequately supplied with water and are unlikely to have been competing for nutrients since the germination medium was pure water. Based on the data in Fig. 1 alone, competition for O_2_ could be a possible explanation for the inhibition of root growth and, at high cress seed numbers, of the hypocotyl growth. However, data in Fig. 2 will refute this explanation.

The batch of cress seeds used in the 2015 experiment proved unusually effective at promoting hypocotyl elongation, but this batch was only moderately effective at inhibiting root growth. This may suggest that the hypocotyl promoter was not identical to the root inhibitor.

### Cress seeds exude a low-molecular weight, hydrophilic, stable ‘allelochemical’

The active principle present in preparations of LCSE, which had been separated from the viscous seed slime by dialysis, was further characterized. The solution of LCSE harvested from outside the dialysis sac typically had a total solute concentration of approx. 1·6 mg mL^–1^. Unmodified LCSE had effects on amaranth seedlings (Fig. 2; compare ‘frozen’ or ‘dried’ with ‘H_2_O’) similar to those of live cress seeds. In the absence of living cress material, competition for dissolved O_2_ is not tenable in this experiment. In a study of the nature of the active principle, aliquots of LCSE were treated with heat, acid and solvent partitioning, and then the bioassay was repeated. Neither heating in solution at neutral pH nor treatment with cold acid had any effect. The active principle partitioned into slightly acidified water in preference to ethyl acetate, showing that it is hydrophilic. All the above observations would be consistent with the proposal that the active principle is LMA. However, hot acid also had very little effect on the bioactivity (Fig. 2), and this observation is not compatible with LMA, whose glycosidic bond would have been hydrolysed by the hot acid (Iqbal *et al.*, 2016).

In an investigation of the susceptibility of the active principle to dry ashing, which would combust all organic material to CO_2_ + H_2_O, LCSE was heated at roughly 400 or 700 °C and its ability to influence amaranth seedling growth was re-tested (Table 1). Ashing in the hottest Bunsen flame abolished the ability of LCSE to promote hypocotyl growth, superficially suggesting that the active principle was organic; however, brief ashing in a milder flame had little effect despite clearly combusting the LCSE (the pale yellow dried LCSE was first charred to form a black tar, and then incinerated to a white ash at 400 °C). Ashing at either temperature abolished the ability of LCSE to inhibit root growth; indeed, the ashed LCSE may have acquired a slight ability to promote root growth (Table 1). These data thus again suggested that the major hypocotyl elongation promoter was not identical to the major root growth inhibitor, and indicated that the latter was organic whereas the former was inorganic (albeit destroyed in the hottest flame – discussed further below).

**Table 1.** Effect of ashing on the ability of low-molecular weight cress-seed exudate (LCSE) to affect amaranth seedling growth

Germination medium	Hypocotyl length (mm ± s.e.)	*P*-value	Root length (mm ± s.e.)	*P*-value
Experiment 1				
Water	13·8 ± 0·6		35·7 ± 1·1	
Frozen/thawed LCSE	32·9 ± 1·6	0·001	26·3 ± 2·4	0.001
LCSE ashed at 700 °C/redissolved	14·8 ± 0·5	n.s.	39·3 ± 1·4	0.05
Experiment 2				
Water	11·4 ± 0·5		25·7 ± 0·7	
Frozen/thawed LCSE	30·6 ± 1·1	≪0·001	18·0 ± 1·2	<0.001
LCSE ashed at 400 °C/redissolved	25·0 ± 1·6	≪0·001	26·9 ± 1·3	n.s.

Eighteen (experiment 1) or fifteen (experiment 2) fresh samples of LCSE were prepared as described in Fig. 2, and used as media for the germination and growth of amaranth seed(ling)s. Aliquots of each LCSE preparation were either frozen and thawed or dried and then ashed in a Pyrex tube (in experiment 1, for 10 min in the hottest part of a Bunsen flame; in experiment 2, for 3 min in a milder flame). The ash samples were redissolved in the original volume of water (all solutions were within the pH range 6·6–6·8). Ten amaranth seeds were sown per Petri dish, and 1 mL of medium was added. After 4·5 d at 25 °C in the dark, the seedlings were measured (there was no effect on percentage germination; data not shown). Data are mean organ lengths ± inter-plate s.e. (*n* = 15 or 18). The *P*-value was calculated by the Student *t*-test in comparison with the corresponding H_2_O control; n.s = not significant (*P* > 0·1).

### The major hypocotyl-stimulating component of LCSE has a very high positive charge:mass ratio

**Fig. 3.**
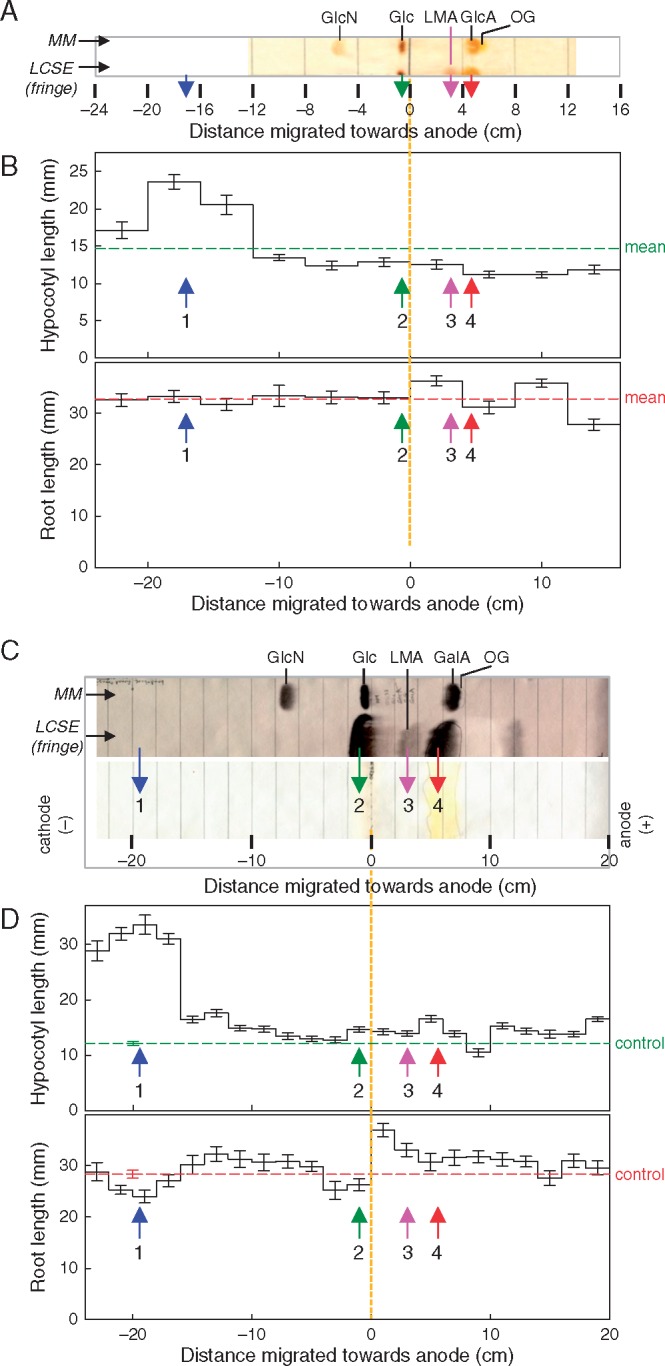
The hypocotyl elongation-promoting material in low-molecular weight cress-seed exudate (LCSE) is strongly cationic and does not co-electrophorese with any detectable carbohydrates. Two independent preparations of LCSE were electrophoresed at pH 6·5 and 3·0 kV for (A) 15 min or (C) 22 min. A marker mixture (MM) plus the fringe of the LCSE track were stained with (A) aniline hydrogen phthalate (revealing reducing sugars) or (C) AgNO_3_ (revealing total sugars). The unstained portion of each LCSE track (only shown in C; containing a trace of orange G as internal marker) was cut into strips as indicated by the pencil lines; each strip was soaked in water, and the eluate was tested in the amaranth bioassay (B, D). In (B), error bars show the inter-dish s.e.; *n* = 13 dishes with ten seedlings per dish; in (D) error bars show the s.e. of 20 individual seedlings grown in two dishes. The dashed lines in (B) represent the mean of the ten 4-cm strips in each run; in (D) they show the measurements obtained in controls with water as the germination/growth medium. Marker abbreviations: GlcN, glucosamine; Glc, glucose; GlcA, glucuronic acid; GalA, galacturonic acid; OG, orange G (7-hydroxy-8-phenylazo-1,3-naphthalenedisulphonic acid). The vertical arrows indicate the positions of (**1**) active principle; (**2**) neutral sugars, e.g. glucose; (**3**) lepidimoic acid (LMA); and (**4**) galacturonic acid.

In a study of the net charge of the active principle, further samples of LCSE were subjected to HVPE in pH 6·5 buffer, and then eluates from strips of the paper were bioassayed. In previously reported runs of this experiment, Iqbal *et al.* (2016) had bioassayed only material that migrated with mobilities in the *m*_GlcN_ ranges –1·9 to + 1·9 or –2·2 to + 1·0 and observed very little biological activity (figs 2 and 3 of Iqbal *et al.*, 2016). [The *m*_GlcN_ of a substance under investigation is its electrophoretic mobility relative to the mobilities of marker glucosamine (*m*_GlcN_ = +1·0) and glucose (*m*_GlcN_ = 0·0); note that glucose, although neutral, moves away from the origin slightly, owing to electro-endo-osmosis.] In the present work, however, the electrophoretic run time was shortened to 15 min so that a wider range of *m*_GlcN_ values could be covered (+4·9 to –3·4; Fig. 3A). As expected, staining a fringe of the electrophoretogram revealed a spot in the position occupied by LMA (Fig. 3A). Under these conditions, the hypocotyl-promoting principle was found as a very rapidly migrating cation (*m*_GlcN_ approx. +3 to + 4; Fig. 3B). This behaviour is clearly incompatible with LMA, which is negatively charged, and indicates a bioactive substance with a positive charge:mass ratio much higher than that of glucosamine.

In experiments designed to confirm and extend these findings, the components of a new batch of LCSE were fractionated by preparative HVPE and then bioassayed (Fig. 3C, D). Authentic sugar markers plus a fringe from the preparative electrophoretogram were stained for total sugars: a moderately anionic sugar was again detected in the position expected for LMA (Fig. 3C), but the major bioactive material was confirmed to be highly cationic (Fig. 3D).

### Potassium ions are the principal growth promoter in LCSE

**Fig. 4.**
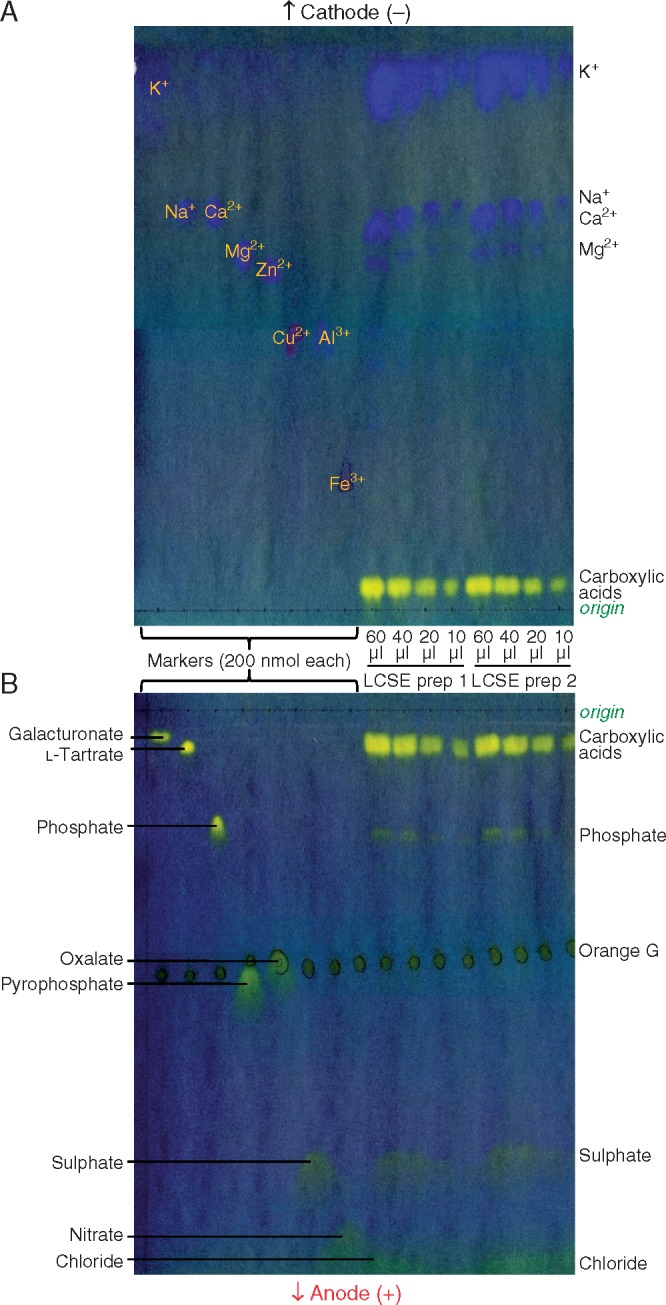
High-voltage paper electrophoresis (HVPE) in pH 2·0 buffer resolves the major strongly cationic and anionic components present in low-molecular weight cress-seed exudate (LCSE). Various volumes (10–60 μL) of 10-fold concentrated LCSE (two independent preparations) were electrophoresed at 3·0 kV in pH 2 buffer alongside authentic markers (0·2 μmol of the named ion in each case). (A) Electrophoresis towards the cathode for 25 min; the markers were loaded as their chloride salts. (B) Electrophoresis towards the anode for 30 min; the markers were loaded as their sodium salts. Each sample contained a trace of Orange G (circled in pencil before staining) as internal marker. The papers were stained with bromophenol blue, revealing cations (blue) and anions (yellow).

Since the data confirmed that LMA is not a strong promoter of hypocotyl growth, and showed that the major growth promoter present in LCSE has a very high positive charge:mass ratio, possible alternative identities of the ‘allelochemical’ were investigated. Ions with very high charge:mass ratios would include inorganics, so the major inorganic ions present in LCSE were surveyed (Fig. 4A). Electrophoresis towards the cathode, in a buffer at pH 2·0, revealed several fast-migrating metal ions co-electrophoresing with K^+^, Na^+^/Ca^2+^ and Mg^2+^ (Fig. 4A). Note that in aqueous solution, K^+^ has a substantially higher effective charge:mass ratio (and thus electrophoretic mobility towards the cathode) than Na^+^; this is because of the smaller hydration shell round K^+^, despite K itself having the higher atomic weight. Electrophoresis towards the anode (Fig. 4B) revealed a heavy spot of carboxylic acids (most of which, except oxalate, are almost un-ionized at pH 2·0 and therefore hardly migrate) as well as three inorganic anions: phosphate, sulphate and chloride. Thus cress seeds released several inorganic ions into the surrounding water.

**Fig. 5.**
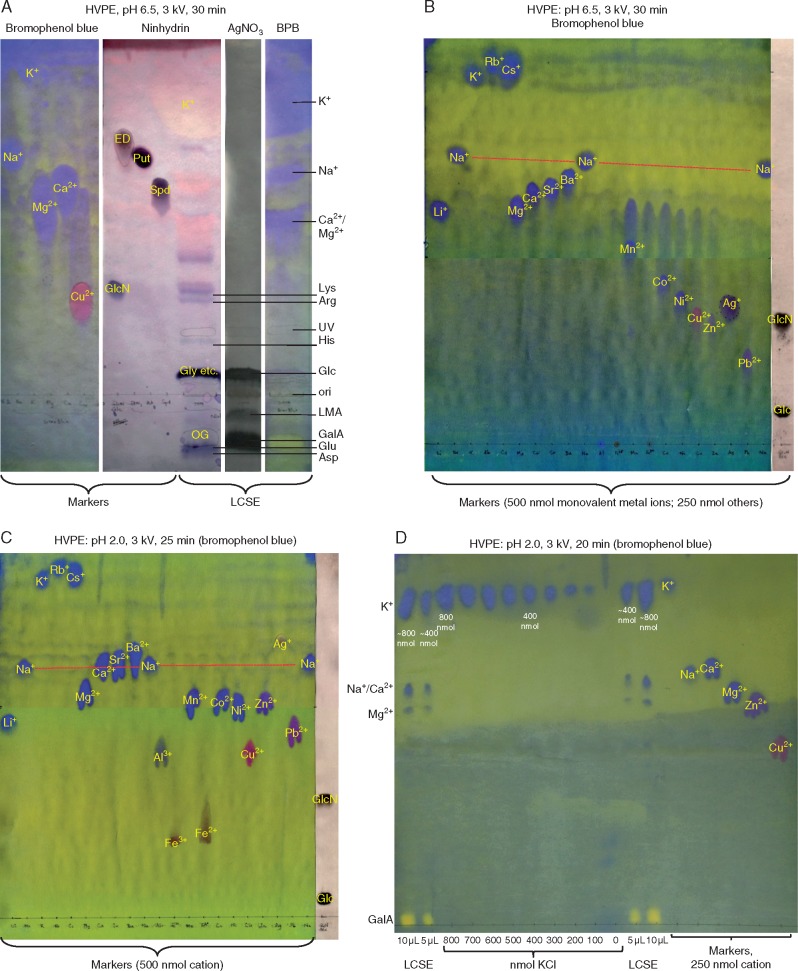
HVPE at two pH values distinguishes K^+^ from all other naturally occurring cations, and shows that K^+^ is present at approx. 4 mm in cress-seed exudate. (A) HVPE of low-molecular weight cress-seed exudate (LCSE) and markers at pH 6·5. Different parts of the same electrophoretogram were stained with bromophenol blue (for inorganic cations), ninhydrin (for amines) or AgNO_3_ (for sugars). Note that non-ionic sugars (e.g. glucose) and amino acids with no net charge (e.g. glycine) drift slightly from the origin owing to electro-endo-osmosis. (B) HVPE of various metal ions at pH 6·5. Fe^2+^, Fe^3+^ and Al^3+^ were not stainable probably because they form insoluble hydroxides at pH 6·5. Na^+^ was loaded at three points, demonstrating uniformity of migration (dashed red line). (C) As (B) but electrophoresis was conducted at pH 2·0. (D) Semi-quantification of inorganic cations in LCSE: 5- and 10-μL aliquots of 20-fold concentrated LCSE were subjected to HVPE at pH 2·0 alongside 250-nmol loadings of selected metal ions and various loadings (0–800 nmol) of K^+^. Non-standard abbreviations: BPB, bromophenol blue; ED, diaminoethane; OG, Orange G; ori, origin (sample loading point); Put, putrescine; Spd, spermidine; UV, spot visible under ultraviolet.

Naturally occurring cations with a substantially higher charge:mass ratio than glucosamine include not only metal cations but also polyamines (Fig. 5A). The presence of several fast-migrating cations in LCSE, including amino compounds and inorganic ions, was indicated by staining after HVPE at pH 6·5 (Fig. 5A). A heavy spot of K^+^ was also found, and the *m*_GlcN_ of K^+^ agreed with that of the active principle (Fig. 3B, D).

**Fig. 6.**
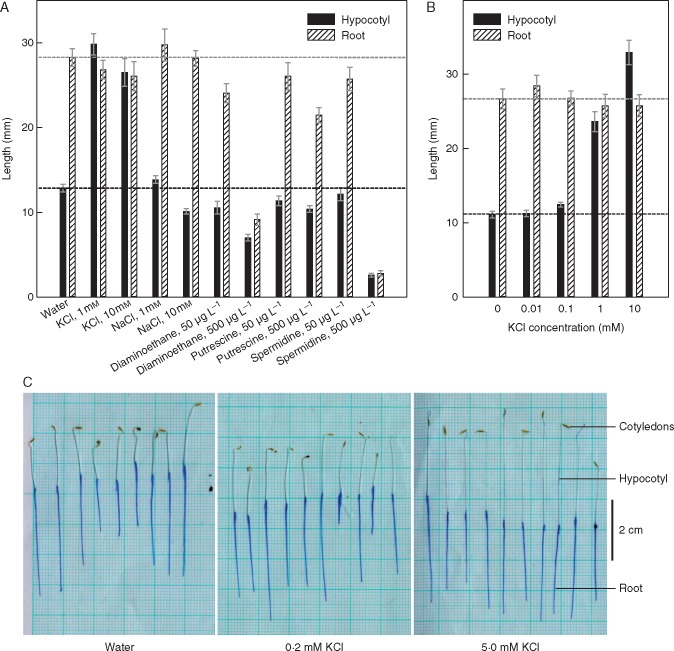
K^+^ at 0·1–10 mM is the only tested cation of high charge:mass ratio that promotes amaranth hypocotyl elongation. Amaranth seeds were sown on filter paper soaked with the solutions indicated. Hypocotyl and root lengths were documented after 4·5 d incubation in the dark. Error bars indicate the s.d. for 20 seedlings grown in two Petri dishes. (A) Various cations, each tested at two concentrations; (B) various concentrations of KCl; (C) appearance of representative seedlings grown in two concentrations of KCl, showing the use of aniline blue to define the boundary between root (stained) and hypocotyl (white). The polyamine solutions were prepared by 10- and 100-fold dilution (into pure water) of 0·5 % (w/v) solutions of the chloride salts of diaminoethane (pH 4·3), putrescine (pH 5·2) or spermidine (pH 4·6).

Several naturally occurring cations were tested for bioactivity on amaranth hypocotyl elongation, and among these only K^+^ was found to be an effective promoter (Fig. 6A), supporting the hypothesis that K^+^ is the major active principle of LCSE. K^+^ had this effect at concentrations between 0·1 mm and at least 10 mm (Fig. 6A). K^+^ strongly promoted hypocotyl elongation but had no significant effect on root growth (Fig. 6C). Na^+^ did not mimic K^+^, even at 10 mm, indicating that the effect was not simply osmotic. Diaminoethane and spermidine inhibited root elongation, but only at concentrations that also strongly inhibited hypocotyl growth. The highest amine concentration tested, 3·9 mm diaminoethane, was of a lower molarity than the K^+^ and Na^+^, indicating that the effect of the amines was not osmotic. Thus, K^+^ is identified as the hypocotyl-promoting principle of LCSE, although it cannot account for the root-inhibiting effect.

The HVPE at pH 2·0 confirmed that K^+^ was the major cation present in LCSE, estimated to have a concentration of approx. 4 mn (Fig. 5D), which is sufficient to be the hypocotyl promoter. Other inorganic cations detected were approx. 0·5 mn Ca^2+^ and/or Na^+^, and approx. 0·3 mn Mg^2+^ (Fig. 5D). The only other cations found to migrate close to the K^+^ zone were Rb^+^ and Cs^+^ (Fig. 5B, C), which are not likely to be present in seeds.

### Effect of dry ashing

**Fig. 7.**
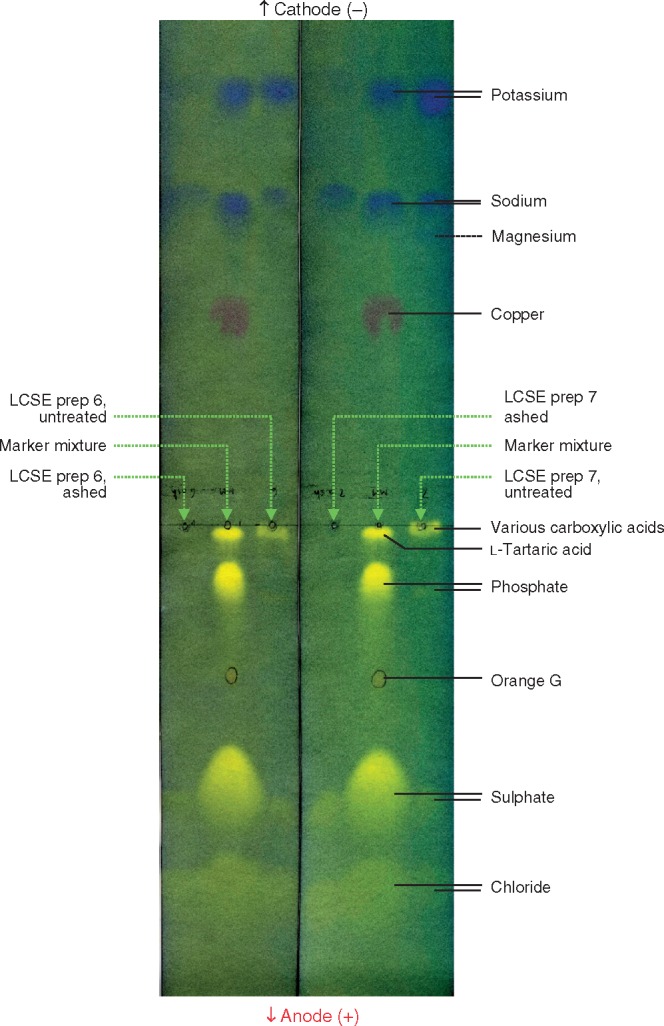
K^+^, like the hypocotyl-promoting principle present in low-molecular weight cress-seed exudate (LCSE), is volatilized on ashing at approx. 700 °C; other inorganics are not volatilized. From two independent preparations (labelled ‘6’ and ‘7’) of 10-fold concentrated LCSE, 100 μL was loaded on the electrophoretogram. In the ashed samples, the residue was re-dissolved in the original volume of water before loading. The marker mixture contained several arbitrarily chosen ions including Orange G. Samples were loaded midway between the anode and cathode, and electrophoresis was conducted at 3·0 kV in pH 2 buffer for 12 min. The papers were stained with bromophenol blue, revealing cations (blue) and anions (yellow).

If the active principle is K^+^, it was initially surprising that the hypocotyl-promoting activity was lost upon dry ashing at about 700 °C (Table 1). However, HVPE showed that LCSE did lose most of its K^+^ during ashing in the hottest part of a Bunsen flame (Fig. 7). Some loss of K^+^ on ashing of *Chamaecyparis obtusa* stems at 600 °C, but not at 400 °C, has been reported before (Koh *et al.*, 1999). Other detectable inorganic ions (Na^+^, Ca^2+^, Mg^2+^, ${\text{SO}}_{4}^{2–}$ and Cl^–^) were not lost by ashing at about 700 °C (Fig. 7). The spot of carboxylic acids completely disappeared after ashing, as expected of organic matter. The loss of hypocotyl-stimulating activity upon ashing at approx. 700 °C, concurring with the loss of detectable K^+^, supports the conclusion that K^+^ is the active principle.

**Fig. 8.**
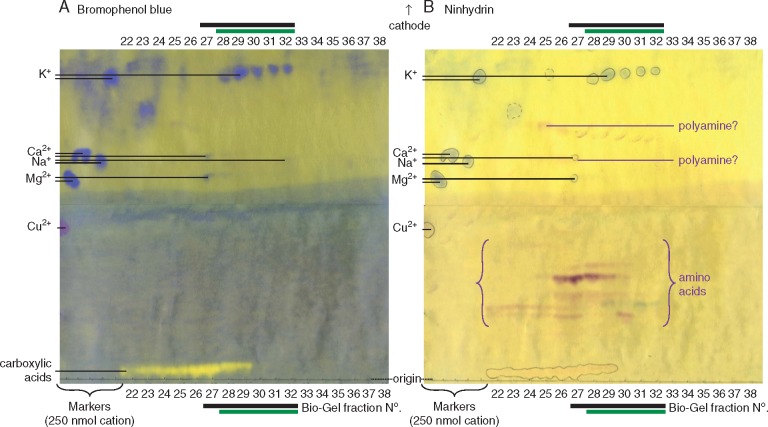
The hypocotyl-promoting factor present in low-molecular weight cress-seed exudate (LCSE) co-elutes with K^+^ (but not Na^+^, Ca^2+^ or Mg^2+^) during gel permeation chromatography; the root-inhibiting principle does not. LCSE was fractionated by gel permeation chromatography on Bio-Gel P-2 (see fig. 4 of Iqbal *et al.*, 2016), then 45-μL aliquots of all relevant fractions were subjected to electrophoresis at pH 2·0 (3 kV, 20 min). Cations were visualized with bromophenol blue (A), then the paper was stained with acidified ninhydrin, revealing more sensitively the amino compounds (B). Black bar, fractions inhibiting root growth; green bar, fractions promoting hypocotyl growth (as reported by Iqbal *et al.*, 2016).

It was previously shown (fig. 4 of Iqbal *et al.*, 2016) that the active principle of LCSE does not co-elute with LMA during gel permeation chromatography on Bio-Gel P-2; instead both the hypocotyl-promoting factor and the root-inhibiting factor eluted between the peaks of sucrose and glucose. In view of the new evidence implicating K^+^ as the hypocotyl-promoting factor, portions of the same Bio-Gel P-2 fractions (kept frozen since the earlier work) were now re-analysed by HVPE at pH 2·0, revealing which fractions contained inorganic K^+^, other inorganic cations and ninhydrin-positive amino compounds (Fig. 8). The peak of the hypocotyl-promoting activity (Bio-Gel fractions 28–32) coincided exactly with the peak of K^+^. The peak of root growth inhibition also covered fractions 28–32 but began in fraction 27, whereas K^+^ did not start eluting until fraction 28 (and K^+^ has little effect on root growth; Fig. 6). Thus, again, the evidence supports K^+^ as the hypocotyl growth promoter and suggests that the root inhibitor is a different substance.

## DISCUSSION

Considerable interest has centred on the report that LMA, an unsaturated acidic disaccharide presumed to be derived from rhamnogalacturonan-I by the action of a lyase, is an allelochemical exuded by seed(ling)s of cress and many other species, and capable of detrimentally influencing the growth of neighbouring, potentially competing, seedlings of other species (Hasegawa *et al.*, 1992; Yamada *et al.*, 1995, 1996, 2007). In the experimental system used for this work, the allelochemical donor species was cress and the model receiver species was *Amaranthus caudatus*. Iqbal *et al.* (2016) had previously confirmed that LMA is exuded by cress seeds, but showed that it does not strongly influence the growth of amaranth hypocotyls or roots. In addition, they showed that at least one other pectin-derived acidic disaccharide [β-d-xylopyranosyl-(1→3)-d-galacturonic acid] is also exuded by cress seeds, but that this too has little if any allelochemical activity (Iqbal *et al.*, 2016). I was therefore interested in elucidating the nature of the true active principle present in cress-seed exudate.

In the present work, the existence was confirmed of heat-stable, hydrophilic, low-molecular weight material, exuded by cress seeds during imbibition, capable of overstimulating amaranth seedling hypocotyl elongation and inhibiting amaranth root growth. The hypocotyl stimulant had a very large, positive, charge:mass ratio, and it co-migrated with K^+^ on HVPE, a procedure which resolved K^+^ from all other known plant substances. K^+^, uniquely among the inorganic cations detected, was lost during dry ashing at about 700 °C in a Bunsen flame; concurrently, the hypocotyl-stimulating activity was lost. These findings strongly point to K^+^ as the major, or sole, hypocotyl stimulant present. Furthermore, it was shown that K^+^ (as KCl) at the concentration occurring in LCSE strongly promoted amaranth hypocotyl elongation. K^+^ was approx. 15-fold more effective than the disaccharide LMA at promoting hypocotyl elongation.

Stimulatory effects of K^+^ on hypocotyl elongation have been reported before, and are probably due to the role of K^+^ as a major osmotic component of cell sap, helping to maintain turgor (Stuart and Jones, 1978; de la Guardia and Benlloch, 1980; McIntyre and Boyer, 1984).

The excessively long and thin hypocotyls developed in the presence of K^+^ may disadvantageously influence seedling establishment, resulting in seedlings that are too weak to withstand damage by the mechanical stresses met in the natural environment. This could benefit the seeds that release K^+^, minimizing competition from neighbouring seedlings. Such a potential benefit has to be weighed against the likely disadvantage of losing an important mineral such as K^+^ into the surrounding soil. However, K^+^ release has been reported before during the early stages of seed imbibition. For example, lupin seeds release K^+^ during the first 4 h, and thereafter may re-absorb it (Scarafoni *et al.*, 2013). Bean (*Phaseolus vulgaris*) seeds also release large amounts of K^+^ during imbibition and germination (Kato *et al.*, 1997). Such release may simply be an inevitable loss from seeds in the early stages of imbibition, before the resumption of active metabolism permits membrane repair.

The present work shows that K^+^ is the major hypocotyl promoter exuded by cress seeds. However, the same seeds also exude a factor that inhibits root growth. Four pieces of evidence together indicate that the root growth inhibitor is different from the hypocotyl promoter: (1) only the former was destroyed by ashing at both 400 and 700 °C (Table 1) and is therefore likely to be organic; (2) in year-to-year variation between batches of cress seeds, high hypocotyl promotion did not correlate with high root inhibition (Fig. 1); (3) there was little or no correlation between the behaviour of the hypocotyl promoter and the root inhibitor during HVPE (Fig. 3); and (4) the two active factors did not precisely co-elute on gel permeation chromatography (Fig. 8). Furthermore, 0·01–10 mm KCl had little effect on root growth while strongly promoting hypocotyl elongation (Fig. 6). The identity of the organic substance(s) responsible for root growth inhibition remains unknown; the observation that its activity is largely lost upon electrophoresis (Fig. 3) suggests that two or more factors may act synergistically and are separated during electrophoresis.

## Conclusions

During imbibition, cress seeds exude ‘allelochemicals’ that overstimulate hypocotyl elongation and inhibit root growth in neighbouring amaranth seedlings. The hypocotyl promoter is shown to be different from the root inhibitor, which remains unidentified. The principal hypocotyl promoter is shown to be K^+^, not the pectic disaccharide LMA as reported before. The exudation of K^+^ by cress seeds into the surrounding soil may be detrimental to the cress, squandering an important reserve nutrient, and/or beneficial, causing the allelopathic overstimulation of stalk elongation in neighbouring competitors.
